# Kikuchi-Fujimoto Disease: Diagnosis in a Relapsing Case

**DOI:** 10.7759/cureus.19542

**Published:** 2021-11-13

**Authors:** Catarina Faria, Marco Fernandes, Rui Cunha, Hugo Moreira, Rui Costa

**Affiliations:** 1 Internal Medicine Department, Hospital São Francisco Xavier, Lisbon, PRT; 2 Internal Medicine Department, Hospital São Francisco Xavier, Lisboa, PRT; 3 Internal Medicine, Hospital da Luz, Lisboa, PRT

**Keywords:** relapsing lymphadenopathy, histologic diagnosis, cervical lymphadenopathy, histiocytic necrotizing lymphadenitis, kikuchi-fujimoto disease

## Abstract

The differential diagnosis of cervical lymphadenopathy is varied. Different age groups require different approaches. Kikuchi-Fujimoto disease or histiocytic necrotizing lymphadenitis is a rare but important diagnosis to consider after excluding more common aetiologies.

We present the case of a 21-year-old female with painful left cervical swelling, lasting over a week. Physical examination revealed multiple cervical lymphadenopathies, elastic, non-adherent to deep tissues which were tender to touch. Blood tests showed elevated acute phase proteins. Cytomegalovirus, Epstein-Barr, toxoplasmosis, and human immunodeficiency virus serologies were negative. Computed tomography of the neck revealed multiple cervical lymphadenopathies which were round-shaped, some with necrosis and with extracapsular extent. These features could be compatible with tuberculous lymphadenitis. However, interferon-gamma release assay was negative. Excisional biopsy was scheduled, but spontaneous regression did not allow it. Two weeks later she relapsed. Excisional biopsy revealed histiocytic necrotizing lymphadenitis.

Kikuchi-Fujimoto’s diagnosis demands high clinical suspicion and histological documentation. This case represents a rare diagnosis of a relapsing disease.

## Introduction

Kikuchi-Fujimoto disease (KFD), or histiocytic necrotizing lymphadenitis, is a rare disease, of unknown etiology, usually benign and with a self-limited course [1,2]. It is more frequent in women, typically between 20 and 40 years old [1]. This disease occurs in all races [3] but it is more prevalent in Asia, particularly in Japan, although the geographical and ethnic correlation has not been completely understood [4]. Clinical presentation includes cervical lymphadenopathy that may or may not be accompanied by fever and other general symptoms [5]. Involvement of the posterior cervical group is the most common, but all areas can be involved [6]. The clinical features are nonspecific and may be compatible with multiple diagnoses. Thus, KFD may be considered after the exclusion of more common causes of lymphadenopathy [6]. Diagnosis of KFD is based on histological findings of the involved lymph nodes. Management is mainly supportive with antipyretics and analgesics [7]. Relapse is rare but possible [8]. We present the case of a 21-year-old female, with a histologic diagnosis possible in relapsing disease. This article was previously presented as a meeting abstract at the 24th Congresso Nacional de Medicina Interna, in June 2018.

## Case presentation

The patient had a past medical history of polycystic ovary syndrome and idiopathic intracranial hypertension headache. She was admitted to the emergency unit with left cervical swelling, associated with pain worsened by movement and alleviated by non-steroidal anti-inflammatories (NSAID), lasting over a week. She also referred to feelings of general malaise and denied having fever, night sweats, anorexia, weight loss, odynophagia, dysphagia, respiratory symptoms such as cough and sputum production, malar rash, photosensitivity, arthritis, alopecia, oral ulcers, or sicca syndrome. She had not traveled recently or had any contact with animals. She did not smoke, drink alcohol, or use illicit drugs. She was a student doing an internship that involved contact with the elderly.

Physical examination revealed left cervical edema with multiple lymphadenopathies in the anterior and posterior cervical chains, enlarged, elastic, non-adherent to deep tissues, and tender to touch on both sides of the neck. There were no other palpable enlarged nodes. The oral cavity showed no alterations. Organomegalies were not noted. Physical examination showed no other relevant findings.

**Figure 1:**
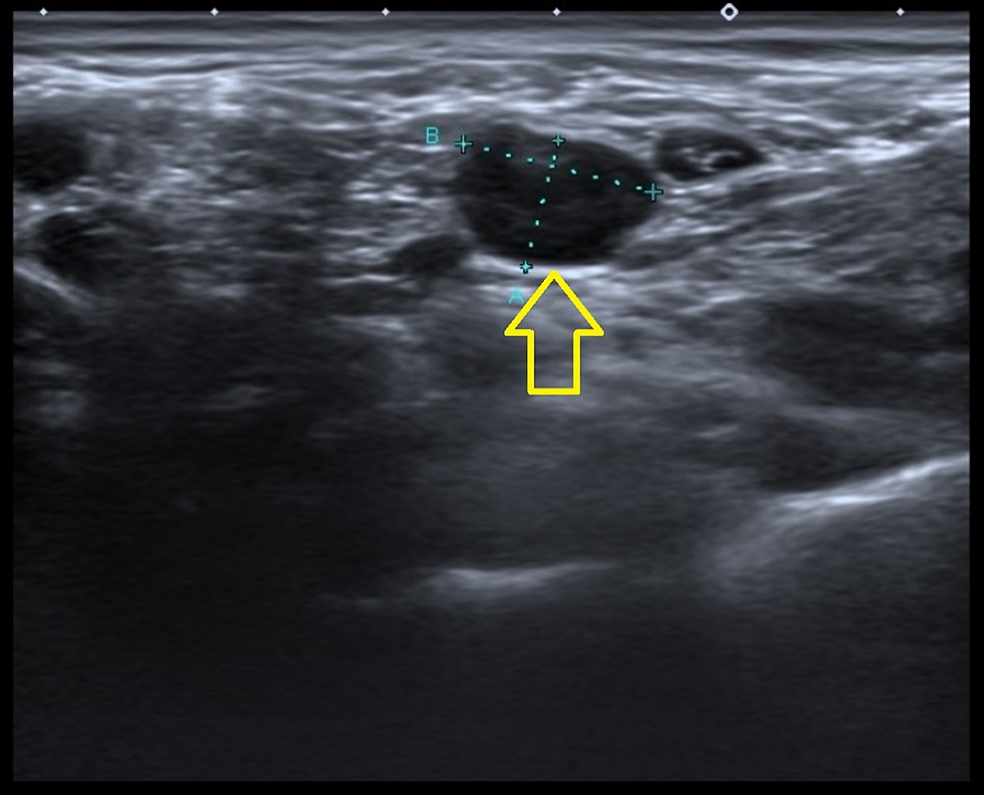
Adenophatic formation bigger than one centimeter Yellow arrow showing adenophatic formation

A blood test showed an erythrocyte sedimentation rate of 58mm/h and C-reactive protein 9.24mg/dL. Hemogram, leucogram, platelet count, kidney and liver function were normal. Ultrasound evaluation showed multiple adenophatic formations in the posterior cervical chain and along the internal jugular chain, with a diameter of over 1 centimeter (Figure 1).

Cytomegalovirus, Epstein-Barr, Toxoplasma gondii, and human immunodeficiency virus serologies, as well as Interferon-gamma release assay, were negative.

**Figure 2:**
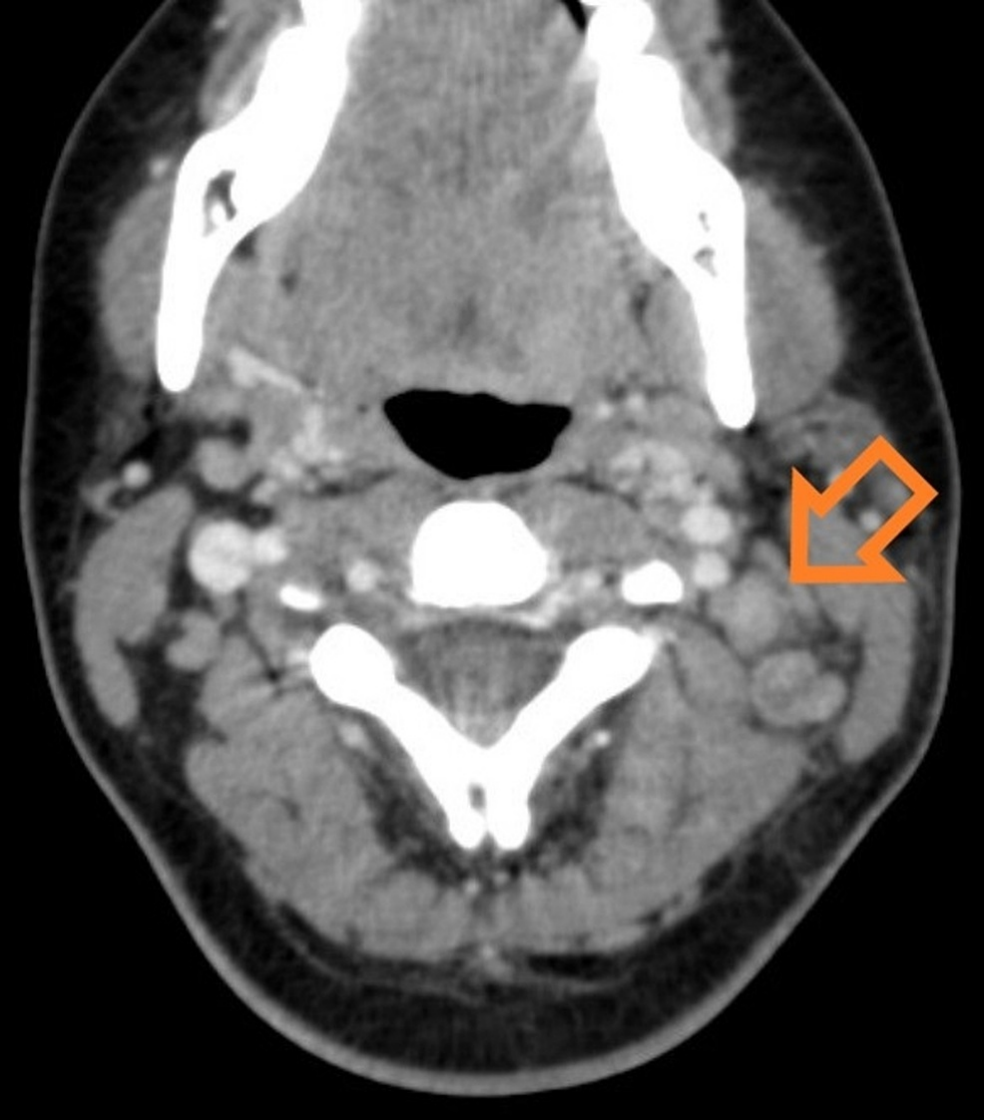
Computed tomography showing multiple cervical lymphadenopathies Orange arrow showing lymphadenopathies

The persistence of the symptoms after excluding common causes, and pathologic appearance on ultrasound, motivated computed tomography of the neck which showed multiple cervical lymphadenopathies, round shaped, some with necrosis and with extracapsular extent, with densification of surrounding fat tissue (Figure 2).

There were no enlarged lymph nodes in the mediastinum, axillar or thoracic chains. These features could be compatible with tuberculous lymphadenitis but no other clinical or laboratory findings supported this diagnosis. Calcium and angiotensin-converting enzyme (ACE) levels were normal. Antinuclear antibodies, anti-dsDNA, anti-Smith antibodies were negative.

It was decided to perform an excisional biopsy. However, spontaneous resolution of the adenopathies made it impossible. She was discharged with a reevaluation scheduled in one week. Two weeks later she relapsed, and an excisional biopsy was performed. Excisional biopsy revealed “lymph nodes with active germinal centers, expansion of the paracortical area with histiocytes and apoptotic bodies with focal necrosis. There were no vasculitic lesions. CMV and EBV were negative. Morphologic aspects compatible with histiocytic necrotizing lymphadenitis.” Based on these results, the diagnosis of Kikuchi-Fujimoto’s disease was made. In time, she had a spontaneous resolution, and no other relapses were recorded during the follow-up time.

## Discussion

Cervical lymphadenopathy differential diagnosis is diverse. Different age groups require different approaches [9]. In young adults, cervical lymphadenopathies are usually the manifestation of inflammation or infection. Most of the time, the clinical presentation is benign and self-limited. Further investigation is needed when the adenopathies persist or pathological features are found [9,10]. Since there were no other enlarged lymph nodes besides those in the neck, local bacterial infection was considered [5]. Careful examination of the oral cavity and paranasal sinus excluded this hypothesis. Given the age of the patient, short course of symptoms and a physical examination with benign characteristics, viral infection was likely [5]. However, CMV and EBV serology were negative for active infection. Pathological changes in ultrasound and neck CT prompted further investigation. Portugal remains a medium-high prevalence country of Mycobacterium tuberculosis, and contact with the elderly represents an important risk factor. However, interferon-gamma release assay was negative and no other signs or symptoms supported this diagnosis.

It was decided to perform an excisional biopsy in order to exclude more serious diseases such as lymphoma, but spontaneous regression of the adenopathies made this impossible. Given this and a normal blood count, we considered lymphoma to be an unlikely diagnosis.

An infectious or inflammatory etiology was the most reasonable explanation. As the patient was stable, she was discharged and requested to return later for a follow-up. After relapsing, an inflammatory disease such as sarcoidosis, or an auto-immune disease such as Systemic Lupus Erythematosus (SLE) should be considered. Chest CT did not reveal lung involvement or any mediastinal or hilar adenopathies. Calcium and ACE were normal, making sarcoidosis a less likely diagnosis [11]. Lymphadenopathy has been reported in 23-34% of patients with SLE. Generally, the nodes are soft and may fluctuate with disease activity [12]. However, she had no other symptoms suggestive of autoimmune disease and there were no laboratory findings that supported that etiology. At this point, she did not meet the necessary criteria for any of these diagnoses. After recurrence of the enlarged neck lymphadenopathies, an excisional biopsy was performed and the diagnosis was made.

KFD is a clinicopathological entity making its diagnosis only possible by compatible histology [5]. It usually affects young women, such as our patient. The clinical presentation with neck lymphadenopathies is typical and may or may not be accompanied by general symptoms, such as malaise as described by the patient. Spontaneous resolution is the rule. We describe a case of relapsing disease which is a rare scenario, occurring in 3-4% of KFD [8]. Supportive care is the main medical intervention, although there are some documented cases of patients with recurrent disease who need treatment with corticosteroids or immunosuppressive drugs [6]. Our patient had a good outcome, needing only NSAID treatment for a short period of time with spontaneous resolution and no other recurrence to date.

## Conclusions

KFD is a rare but important diagnosis to consider after excluding the more common etiologies of cervical lymphadenopathy. This case represents a rare diagnosis of relapsing disease. The diagnosis needs high clinical suspicion and histological documentation. Even though some cases need more aggressive treatment, it is usually benign and self-limited. Clinicians should remember this disease and wait for the final diagnosis if no other criteria are found before initiating immunosuppressive therapies that may lead to avoidable side effects and complications.
